# Ovarian mucinous tumors with mural nodules: immunohistochemical and molecular analysis of 3 cases

**DOI:** 10.1186/s13000-020-00956-6

**Published:** 2020-04-14

**Authors:** Ying Shao, Qin Liu, Haiyan Shi, Bingjian Lu

**Affiliations:** 1 Department of Surgical Pathology, Women’s Hospital, School of Medicine, Zhejiang University, Hangzhou, Zhejiang Province China; 2 Center for Uterine Cancer Diagnosis & Therapy Research of Zhejiang Province, Women’s Hospital, School of Medicine, Zhejiang University, Hangzhou, Zhejiang Province China

**Keywords:** Ovary, Mucinous tumor, Mural nodule, Sarcoma, *K-RAS* mutation, LOH

## Abstract

**Background:**

Primary ovarian mucinous tumors with mural nodules are very rare. The histogenesis of the mural nodules remains unclear.

**Methods:**

We investigated the clincopathological and molecular features in 3 cases with mural nodules.

**Results:**

Patient 1 was diagnosed as mucinous carcinoma with mural nodules of anaplastic carcinoma that was composed of CK+ and CK7+ spindled cells and polygonal cells with marked pleomorphism. Aberrant p53 staining was found in the mural nodules rather than in the mucinous components. A concordant *KRAS* mutation (c.35G > A p.G12A) was identified in both mucinous tumors and mural nodules. She died of disease at 44 months. The mural nodule in patient 2 was interpreted as a sarcoma, no other specified. The uniform short spindle cells were separated by abundant myxoid matrix. They were CD10 + , CCND1-, SMA-, and negative for break-apart *BCOR*, *PHF1*, and *JAZF1* FISH assay. The adenocarcinomatous component harbored LOH at *D18S51* and *FGA loci* while the sarcomatous component had LOH at *D19S433*. She had lung metastasis at 18 months and was alive without evidence of disease for 40 months. Patient 3 harbored multiple mural nodules that were composed of vimentin+, focal CK+, atypical spindle cells. A diagnosis of sarcoma-like mural nodules was rendered. She was alive with no evidence of disease for 13 months. No hotspot mutant *AKT1*, *KRAS*, *HRAS*, and *PI3KCA* alleles were found in patients 2 and 3.

**Conclusions:**

Mural nodules with anaplastic carcinoma or with true sarcomas may represent the dedifferentiation form of mucinous tumors or collision tumors, respectively. The worrisome histology in sarcoma-like mural nodules necessitates meticulous treatment for these patients.

## Introduction

Mural nodules can occur in ovarian mucinous tumors, usually in borderline tumors and adenocarcinomas, but they are very uncommon [1]. A wide variety of mural nodules have been described, such as anaplastic carcinomas, true sarcomas, and sarcoma-like mural nodules (SLMN). Mucinous tumors with malignant mural nodules (anaplastic carcinomas and sarcomas) tend to occur in older patients, and to have a poor clinical outcome; therefore, they are best regarded as the variants of carcinomas or carcinosarcomas [2]. Patients with benign (mainly sarcoma-like) nodules tend to be younger and are believed to be benign, but should be treated with caution because of their worrisome morphology and very limited follow-up data to date [1, 3, 4].

The histogenesis of the mural nodules in ovarian mucinous tumors remains unclear. It is controversial whether they may represent a divergent differentiation (dedifferentiation) of the mucinous tumors or an independent origin (collision tumor) [5]. Molecular investigation may contribute to resolving this issue: the molecular similarities between the mural nodules and mucinous tumors are indicative of a common histological origin while the differences are not. Mutations in *K-RAS* and *PIK3CA* have been frequently found in ovarian mucinous borderline tumors and carcinomas [6–8]. However, they have only been investigated on a very limited number of ovarian mucinous tumors with anaplastic carcinoma mural nodules to date [5, 9, 10]. In this study, we analyzed the *K-RAS*, *PIK3CA*, *AKT1* and *H-RAS* gene mutation status in both components of epithelial elements and mural nodules in ovarian mucinous tumors from 3 patients.

## Patients and methods

Appropriate research permissions were obtained from the hospital’s Institutional Review Board (IRB20170138). Three ovarian mucinous tumors with mural nodules were collected from the institutional database, Women’s Hospital, School of Medicine, Zhejiang University, China, between 2008 and June 2018. The clinical and follow-up data were obtained from chart review and telephone communication. Tumor stage was determined retrospectively according to the International Federation of Gynecology and Obstetrics (FIGO) system (2009). The archival hematoxylin-and-eosin (H&E) slides were re-assessed by two authors (SH & LB).

Four-μm thick formalin-fixed paraffin-embedded (FFPE) slides from both ovarian mucinous tumors and mural nodules were cut for immunohistochemistry. A two-step En Vision immunostaining procedure (DAKO, Carpentaria, USA) was performed according to the manufacturer’s protocols. The panel of diluted antibodies included cytokeratin (CK) (AE1/AE3; DAKO, Carpentaria, USA; 1:100), cytokeratin 7 (CK7) (OV-TL 12/30; Genemed Biotechnologies Inc., Torrance, USA; 1:100), ALK (5A4; Genscript Biotech, Nanjing, China; 1:100), inhibin (EP378; Abcam, Cambridge, UK; 1:25), cytokeratin 20 (CK20) (KS20.8; Leica Biosystems, Nussloch, Germany; 1:100), estrogen receptor (ER) (SP1; Thermo Fisher Scientific, Waltham, USA; 1:300), progesterone receptor (PR) (SP2; Thermo Fisher Scientific, Waltham, USA; 1:500), epithelial membrane antigen (EMA) (E29; Spring Bioscience, Pleasanton, USA; 1:300), Paired Box 8 (PAX8) (GR002; Zeta Bioscience, Shanghai, China; 1:200), caudal type homeobox 2 (CDX2) (EP25; Abcam, Cambridge, UK; 1:100), SALL4 (6E3; Maxim Biotech, Fuzhou, China;1:200), vimentin (V9; Genemed Biotechnologies Inc., Torrance, USA; 1:200), α-smooth muscle actin (SMA) (1A4; Novus Biologicals, Centennial, USA; 1:100), desmin (D33; DAKO, Carpentaria, USA; 1:100), carcinomatous embryonic antigen (CEA) (polyclonal; DAKO, Carpentaria, USA; 1:1000), S-100 (polyclonal; DAKO, Carpentaria, USA; 1:1000), p63 (4A4; Abcam, Cambridge, MA, USA; 1:200), CK5/6 (D5/16B4; Abcam, Cambridge, MA, USA; 1:200), cytokeratin low-molecular weight (CK-LMW) (Cam5.2; DAKO, Carpentaria, USA; 1:50), Wilms-tumor 1 (WT1) (6F-H2; Thermo Fisher Scientific, Waltham, USA; 1:100), CD34 (QBEnd 10; DAKO, Carpentaria, CA, USA; 1:100), CD10 (56C6; dilution 1:100), cyclin D1 (SP4; Thermo Fisher Scientific, Waltham, USA; 1:50), Ki-67 (MIB1; Thermo Fisher Scientific, Waltham, USA; 1:400) and p53 (DO-7; Thermo Fisher Scientific, Waltham, USA; 1:300). The percentage of positive cells < 5, 5–24%, 25–49% and ≧50% was interpreted as negative, weak, moderate and strong staining, respectively. Strong nuclear staining (> 70% cells) or null staining were defined as aberrant p53 expression (mutant type), and otherwise as normal expression (wild type).

Dual color break-apart fluorescence in situ hybridization (FISH) using probes flanking *BCOR* (LBP Med Sci & Tech, Guangzhou, China), *PHF1* and *JAZF1* (ZytoVision GmbH, Cologne, Germany) were performed in patient 2 according to the manufacturer’s protocol. Two hundred nuclei of the tumor cells with the entire visualized nuclear membran were counted. Break-apart signals in ≥10% of cells were considered to represent rearrangement.

Hotspot mutant *AKT1* (E17K), *KRAS* (G12V, G12A, G12D, G13D), *HRAS* (Q61H), and *PI3KCA* (E542K, E545K, E546K) alleles were detected by pyrosequencing, a simple, robust, and sensitive method with a limit of approximately 5% mutant alleles even in paraffin embedded tissues [11]. Each component of ovarian mucinous tumors and mural nodules was manually micro-dissected from the formalin-fixed, paraffin-embedded slides. Genomic DNA was extracted by QIAamp DNA FFPE tissue Kit (Qiagen, Valencia, CA, USA). DNA concentration was measured by the Nanodrop Microliter spectrophotometer (Thermo Fisher Scientific, Australia). Polymerase chain reaction (PCR) and pyrosequencing were performed in a volume of 25 μL reaction system containing ~ 100 ng genomic DNA, and 13 μL 2 × universal mixture (Vazyme, Nanjing, China). The specific PCR steps included 94 °C 5 min, 37 amplification cycles (94 °C 30s, 56–57 °C 30s, 72°C30s), and 72 °C 7 min. The biotinylated PCR products (15 μL) were used for pyrosequencing (Q24 Pyrosequencer, Qiagen GmbH, Hilden, Germany). Gene mutations were identified as per the manufacturer’s protocol. The primer sequences of specific genes have been applied in our study recently [12].

Fluorescence multiplex PCR-capillary electrophoresis (FM-CE) was performed to detect microsatellite instability (MSI) and loss of heterozygosity (LOH) in each component of ovarian mucinous tumors and mural nodules (patient 2) by the commercially available AGCU Express Marker 22 Fluorescence Kit (AGCU Scien Tech Incorporation, Wuxi, China). The kit can detect 22 short tandem repeats (STR) loci that were labeled with four fluorescent dyes (FAM, HEX, TAMRA and ROX). The fluorescent multiplex PCR was performed with GeneAMP PCR system 950 (Applied Biosystems, Foster City, CA). Fluorescently labeled 1 μL PCR products were mixed with 0.5 μL AGCU Marker-500 (internal control) and 9.5 mL Hi-Di Formamide. The mixture was denatured at 95 °C for 3 min, kept at 4 °C for 5 min, and then was detected using the ABI 3130 Genetic Analyzer and GeneMapperID 3.1 software. A direct comparison of the amplicon lengths between the tumors and normal endometrium/myometrium was made for each locus. A specific locus with an amplicon shift (shortening or prolongation of the DNA sequence) or a novel amplicon was regarded as microsatellite instability positive (MSI+). Loss of heterozygosity (LOH) was defined as the allele ratio (the ratio of 2 alleles in cancers vs. that in normal tissues < 0.6 or ≧1.67).

## Results

The clinicopathological and molecular findings of the patients are summarized in Tables 1 and 2, respectively.

**Table 1:** Clinicopathological features of ovarian mucinous tumors with mural nodules

	Patient 1	Patient 2	Patient 3
**Age (yr)**	21	41	27
**G & P**	G0P0	G1P1	G0P0
**Clinical presentation**	abdominal enlargement for 6+ yr	pelvic mass for 9 yr	pelvic mass for 2 mo
**Surgery**	ARSO, omentectomy, appendectomy, PLND	TAHBSO, omentectomy, PPLND	ALSO, omentectomy, appendectomy, removal of right ovarian cyst
**Tumor location**	right ovary	left ovary	left ovary
**Tumor size**	20 × 20 × 19 cm	11 × 10 × 9 cm	40 × 30 × 25 cm
**Mural nodules (number, size)**	2 (4 × 5 × 5 cm and 3 × 1 × 0.8 cm)	2 (3 cm and 5 cm in diameters)	multiple (1-4 cm in diameters)
**FIGO stage**	Ia	IIa	Ia
**Pathological diagnosis**	WDMAC with mural nodules of anaplastic carcinoma	MBT with focal AC and mural nodules of sarcoma with myxoid changes, no other specified	MBT with focal WDMAC and SLMNs
**Concurrent pathology**	N	uterine adenomyosis; right OEC	N
**Adjuvant chemotherapy**	PC*2	TP*6	N
**Follow up (time)**	DOD (44mo)	lung metastasis (18mo); ANED (40mo)	ANED (13mo)

*Abbreviations*: *AC* adenocarcinoma, *ALSO* abdominal left salpingo-oophorectomy, *ANED* alive with no evidence of disease, *ARSO* abdominal right salpingo-oophorectomy, *DOD* died of disease, *G & P* gestation and parity, *mo* month(s), *OEC* ovarian endometriotic cyst, *PLND* pelvic lymph nodes dissection, *PPLND* pelvic/para-aortic lymph nodes dissection, *SLMN* sarcoma-like mural nodules, *WDMAC* well differentiated mucinous adenocarcinoma; yr year(s)

**Table 2:** Immunohistochemical and molecular findings of ovarian mucinous tumors with mural nodules

	Patient 1		Patient 2		Patient 3	
	Mucinous tumor	Mural nodule	Mucinous tumor	Mural nodule	Mucinous tumor	Mural nodule
**Pathological diagnosis**	WDMAC	Anaplastic carcinoma	MBT, focal AC	Sarcoma	MBT, focal WDMAC	SLMN
**Immunostaining**						
CK, CK7	+++	++ ~ +++	+++	–	+++	+/−
EMA, CK20, CEA, CDX2	+	–	+	–	+	–
PAX8, ER, PR	–	–	–	–	–	–
CD10	–	–	–	+++	–	+
SMA	–	–	–	–	–	+
P53	WT	MT	WT	WT	WT	WT
Ki67 index	40%	80%	30%	80%	40%	50%
**Hotspot mutations**						
*KRAS*(G12V, G12A, G12D, G13D)	G12A	G12A	–	–	–	–
*HRAS* (Q61H)	–	–	–	–	–	–
*AKT1* (E17K)	–	–	–	–	–	–
*PI3KCA* (E542K, E545K, E546K)	–	–	–	–	–	–

*Abbreviations*: *AC* adenocarcinoma, *MBT* mucinous borderline tumor, *MT* mutant-type, *SLMN* sarcoma-like mural nodules, *WDMAC* well differentiated mucinous adenocarcinoma, *WT* wild-type

### Clinicopathological findings

#### Patient 1: clinical findings

A 21-year-old Chinese woman presented with gradual abdominal enlargement for more than 6 years. A 30 cm abdominal cystic mass was found on physical examination. Both abdominal-pelvic sonogram and computed tomography (CT) scan showed a large ovarian mass with two intra-cystic solid protrusions. Her serum CA125, CEA, CA153, and alpha-fetoprotein (AFP) were within the normal range. The patient underwent fertility-sparing staging surgery including abdominal right salpingo-oophorectomy, omentectomy, appendectomy, and pelvic lymphadenectomy. The tumor was staged as FIGO Ia (2009). She received two courses of CP (cisplatin + cyclophosphamide) chemotherapy postoperatively. She died of disease at 44 months following her surgery.

#### Patient 1: pathological findings

**Fig. 1:**
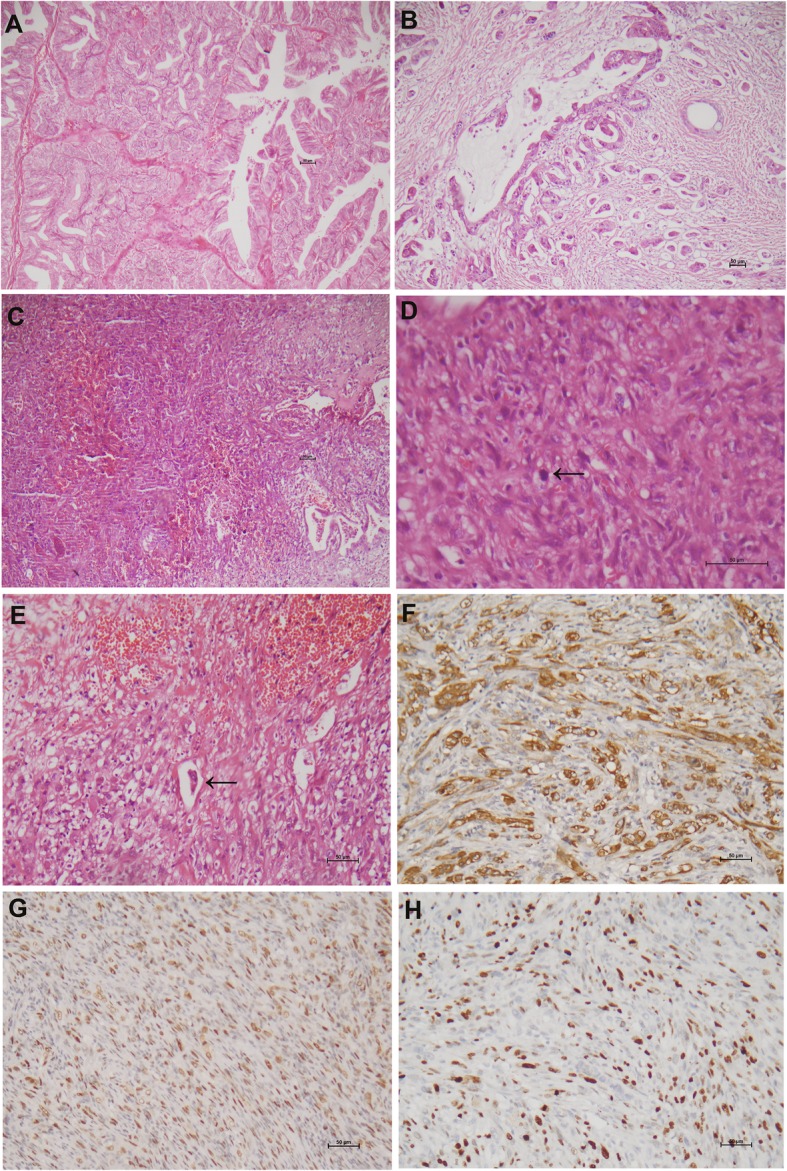
Ovarian well-differentiated mucinous adenocarcinoma with mural nodules of anaplastic carcinoma (patient 1). Well differentiated mucinous carcinoma has an expansile [**a**] and destructive [**b**] invasive pattern. Depicted in **c** & **d** are the mural nodules of anaplastic carcinoma that are composed of spindled cells and polygonal cells with marked nuclear pleomorphism and active mitotic figure. [**d**, arrow]. The presence of atypical glands [right lower in C] is indicative of the transitional zones of the mural nodules. Lymphovascular invasion is shown in E [arrow]. The tumor cells in the mural nodules have a strong CK [F], an aberrant p53 expression [G], and a high Ki67 index [H]. (H&E: A-C × 100, D × 400, E × 200; immunohistochemistry, F-H × 200)

The mass of the right ovary measured 20 × 20 × 19 cm with a smooth outer surface. The cut surface showed a unilocular cyst containing approximately 3000 mL brownish gelatinous fluid. The capsule was 0.4–0.8 cm thick. The inner surface had a velvet-like looking in most areas except 1 papillation and 2 mural nodules. The papillation measured 3.5 × 2 × 1.5 cm with a white and soft cut surface. The 2 mural nodules measured 4 × 5 × 5 cm and 3 × 1 × 0.8 cm, respectively. Both nodules were grayish, solid with hemorrhagic and necrotic areas. Microscopic examination of the right ovarian mass showed a primary ovarian intestinal-type mucinous neoplasm composed of benign, borderline, and invasive areas. The well differentiated mucinous carcinoma was characterized by a confluent growth of interconnecting papillae (expansile stromal invasion) (Fig. 1a) and areas of infiltrative atypical glands, small cords/nests, and single cells in the desmoplastic background or surrounded by a clear space (destructive invasion) (Fig. 1b). The mural nodules were composed of spindled cells and polygonal cells with eosinophilic cytoplasm, marked nuclear pleomorphism and active mitotic figures (~ 12 /10HPFs), imperceptibly merged with the desmoplastic stroma with occasional epulis-like multinucleated giant cells (Fig. 1c, d). Lymphovascular invasion was present in the mural module (Fig. 1e). Occasional atypical glands were detected within the transitional zones of the mural nodules (Fig. 1c). The fallopian tubes, omentum and pelvic lymph nodes were unremarkable.

Immunohistochemistry demonstrated that both components of mucinous tumor and mural nodule had a moderate-to-strong staining for CK (Fig. 1f) and CK7 (Table 2). EMA, CK20 and CEA were positive in the mucinous tumor but negative in the mural nodules. The anaplastic cells in the mural nodules showed an aberrant p53 expression pattern and a high Ki67 index (> 80%) (Fig. 1g, h) whereas the mucinous tumor harbored a wild type p53 staining and a relatively lower Ki67 index (~ 40%). A panel of other antibodies, such as PAX8, CD34, SMA, S100, desmin, ALK, ER and PR, were negative in both components. The epulis-like multinucleated giant cells were positive for CD68 and negative for CK.

The final histopathological diagnosis of the right ovary tumor was a well-differentiated mucinous adenocarcinoma with mural nodules of anaplastic carcinoma arising in a background of mucinous cystadenoma and borderline tumor.

#### Patient 2: clinical findings

A 41-yr-old woman complained of a pelvic mass that had been found 9 years before, and grew rapidly recently. Physical examination revealed a 10 cm pelvic mass. Magnetic resonance image (MRI) scan identified a 10.5 × 8.5 × 11.8 cm cyst with a 4.5 × 5 × 5.5 cm hyperdense area in the cystic wall of the left ovary. Her serum CA125 and AFP level was 58.3 U/mL (normal range ≤ 35 U/mL) and 11.5 ng/mL (normal range ≤ 7.0 ng/mL), respectively. She underwent a staging surgery including total abdominal hysterectomy with bilateral salpingo-oophorectomy (TAHBSO), omentectomy, pelvic and para-aortic lymphadenectomy. The intra-operative findings included a large cystic mass in the left ovary that was adhered to the fallopian tube and the left pelvic wall. No other abnormalities were found in the abdominal and pelvic organs. The tumor was staged as FIGO IIa. Six courses of TP (paclitaxe and carboplatin) chemotherapy were given post-operatively. A recurrent solitary tumor was found in the left lower lobe of the lung 18 months after her surgery. She underwent left inferior lobectomy of the lung and bronchial lymphadenectomy in another hospital. She has been alive with no evidence of disease for 40 months after her first surgery by now.

#### Patient 2: pathological findings

**Fig. 2:**
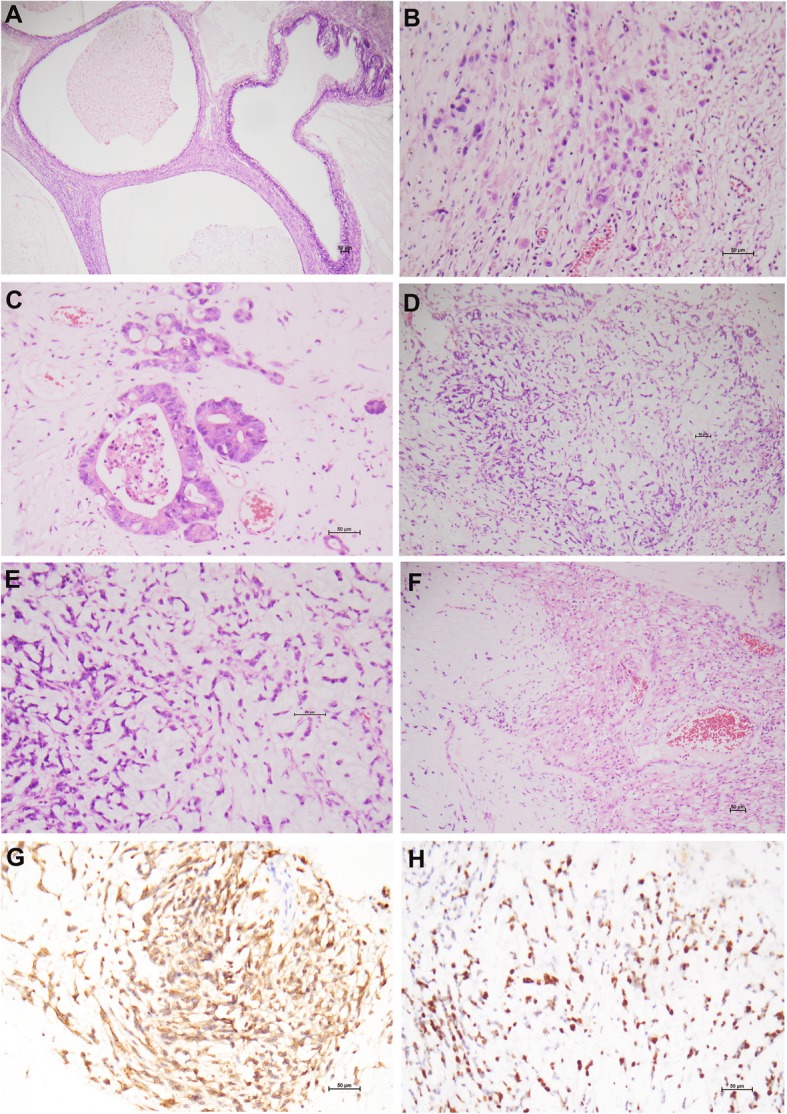
Ovarian mucinous tumor with sarcomatous mural nodules (patient 2). The adenofibromatous areas contain atypical intestinal-type mucinous glands with stratification in keeping with a borderline tumor [**a**]. The invasive lesions are characterized by dyscohesive atypical cells [**b**] and atypical mucinous glands admixed with the myxoid components [**c**]. The mural nodules are composed of uniform short spindle cells separated by abundant myxoid matrix [**d**]. The hyperchromatic tumor cells harbor a high nuclear-cytoplasmic ratio [**e**]. Focal spindle cells with esinophilic cytoplasm arranged in fascicles were suggestive of smooth muscle differentiation [**f**]. The neoplastic cells in the mural nodules are CD10+ [**g**] and have a high Ki-67 index [H]. (H&E: **a** × 5, **b**, **c** & **e** × 200, **d** & **f** × 100; immunohistochemistry, **g** & **h** × 200)

The left ovarian cystic tumor measured 11 × 10 × 9 cm. Two mural nodules measured 3 cm and 5 cm in the largest dimensions, respectively. Both nodules had a gelatinous cut surface. The large nodule was adhesive with the fallopian tube. The remaining capsular wall was 2-4 mm thick. Histopathological assessment indicated a cystic intestinal-type mucinous tumor with adenofibromatous areas containing some atypical mucinous glands with stratification in keeping with a borderline tumor (Fig. 2a). The invasive lesions were manifested as focal atypical esinophilic cells in dyscohesive sheets or singly in the stroma and atypical mucinous glands admixed with the myxoid components (Fig. 2b, c). The mural nodules were composed of uniform short spindle cells arranged in lobules and separated by abundant myxoid stroma (Fig. 2d). The tumor cells harbored a high nuclear-cytoplasmic ratio. The nuclei were hyperchromatic (Fig. 2e). The mitotic figures were 4–6/10HPs. Foci of spindle cells with esinophilic cytoplasm (SMA+) arranged in fascicles were suggestive of smooth muscle differentiation (Fig. 2f). A delicate network of small blood vessels was variably seen. Inflammatory cells were occasionally present. Hemorrhage and tumor necrosis were not uncommon. No lymphovascular invasion was found. The myxoid tumor components involved the fimbria and muscular wall of left fallopian tube. There was no reliable evidence of endometriosis in the left ovary except the foci of hemosiderin-laden macrophages after meticulous examination. The recurrent pulmonary tumor showed the morphology of myxoid sarcoma in line with a metastasis from the ovarian tumors.

The neoplastic cells in the mural nodules were positive for CD10 (Fig. 2g) and CD99, and negative for a panel of antibodies including cyclin D1, ALK, PAX8, CK7, CK20, EMA, CEA, CDX2, ER, PR, SMA, myogenin, S100, HMB45, WT1, inhibin and SALL4. The Ki-67 was approximately 80% (Fig. 2h). The glandular components were diffusely positive for CK7 and negative for CK20 (Table 2). A break-apart FISH assay for *BCOR*, *PHF1*, and *JAZF1* was negative in all components of the ovarian tumors.

The histopathological diagnosis of the left ovary was mucinous borderline tumor with focal invasive adenocarcinoma and mural nodules of sarcoma, no other specified, with myxoid changes invading the left fallopian tube. The concurrent pathology included uterine adenomyosis and right ovarian endometriotic cyst.

#### Patient 3: clinical findings

A 27-yr-old Chinese woman complained of abdominal distension for 6 months. Physical examination indicated a pelvic mass. Both ultrasonography and abdominopelvic computerized tomography (CT) scan showed a large ovarian cyst (approximately 40 cm in the diameter) with multiple mural nodules. The serum CA125 and CA199 levels were 42.3 U/mL and 55.4 U/mL (normal range ≤ 37 U/mL), respectively. The tumor was staged as FIGO Ia. She underwent fertility-sparing staging surgery including abdominal left salpingo-oophorectomy, omentectomy, pelvic lymphadenectomy and appendectomy. She received no adjuvant therapy. She has been unremarkable for 13 months after her surgery at present.

#### Patient 3: pathological findings

**Fig. 3:**
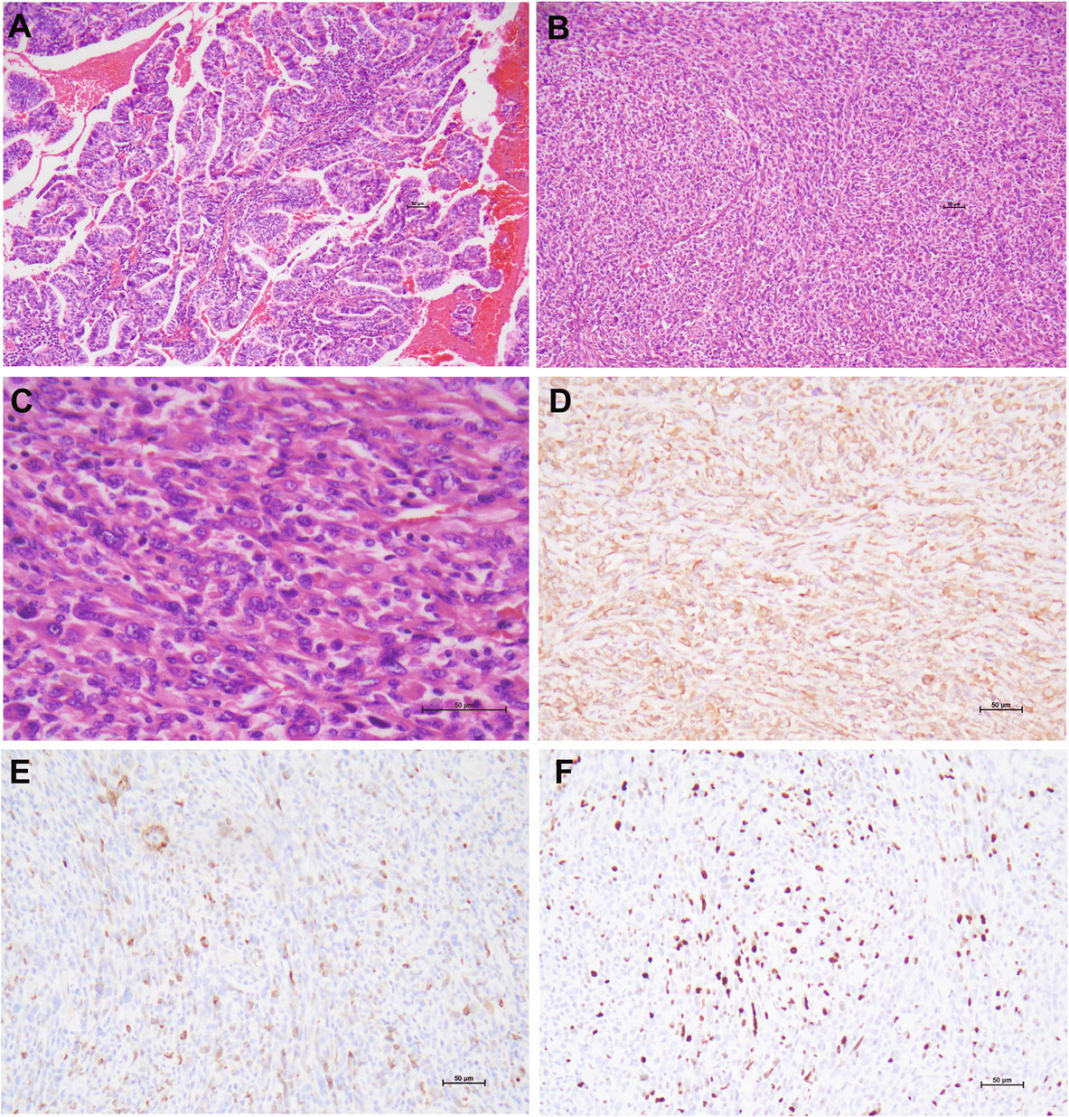
Ovarian mucinous tumor with sarcoma-like mural nodules (patient 3). A well differentiated mucinous adenocarcinoma exhibits the confluent growth of interconnecting papillae [**a**]. The mural nodule is composed of atypical spindle cells arranged in a vaguely fascicular pattern [**b**, **c**]. The tumor cells in the mural nodule are vimentin+ [**d**], CK focal+ [**e**], and have a high Ki-67 index [**f**]. (H&E: **a**, **b** × 100, **c** × 200; immunohistochemistry, **d**-**f** × 200)

Grossly, the intact ovarian mass measured 40 × 30 × 25 cm. It contained 1500 mL brownish gelatinous fluid. The outer surface was smooth. On cut section, it was unilocular with multiple mural nodules. The nodules ranged in 1 to 4 cm in the diameters, and had a whitish, reddish or grayish cut surface. The fallopian tubes, omentum and pelvic lymph nodes were unremarkable. Microscopic examination of the left ovarian mass showed an intestinal-type mucinous neoplasm composed of benign and borderline areas. The confluent growth of interconnecting papillae (about 1 cm in the diameter) suggested a well differentiated adenocarcinoma (Fig. 3a). The mural nodules were well circumscribed. They were predominantly composed of spindle cells with hyperchromatic nuclei and inconspicuous nucleoli arranged in a vaguely fascicular pattern (Fig. 3b, c). The mitotic figures were frequently present (4–5/10HPFs). There were variable histiocytes, lymphocytes, neutrophils and occasional multiple nucleated giant cells. Hemorrhage was frequently seen. No necrosis or vascular space invasion was found.

The glandular components had a strong immunostaining for CK, EMA, CK7, and focal for CK20 and CDX2 (Table 2). The sarcoma-like components in the mural nodules were strongly positive for vimentin (Fig. 3d), focally positive for CK (Fig. 3e), CK7, CD10, and SMA, negative for CEA, Cam5.2, CK5/6, p63, CDX2, CK20 and EMA, a wild-type p53 staining, and a high Ki67 index(~ 50%) (Fig. 3f). Both components were negative for ER, PR, PAX8, HNF1β, napsin A, AFP, CD34, ALK, inhibin, Glypican-3, and SALL4.

The histopathological diagnosis of the left ovary was mucinous borderline tumor with focal carcinomatous changes (well differentiated adenocarcinoma) and SLMNs.

### Molecular findings

The results of mutation analysis are shown in Table 2. A concordant *KRAS* mutation (c.35G > A p.G12A) was identified in both mucinous tumors and mural nodules from patient 1. No other hotspot mutant *AKT1* (E17K), *KRAS* (G12V, G12D, G13D), *HRAS* (Q61H), and *PI3KCA* (E542K, E545K, E546K) alleles were found in both components from 3 patients.

In patient 2, STR analysis failed in 5 loci (*D7S820*, *D2S414*, *Penta D*, *D10S1248* and *D6S1043*). The adenocarcinomatous and sarcomatous component had LOH at *D18S51* and *FGA loci*, and at *D19S433 locus*, respectively. No MSI was detected in either of both components.

## Discussion

Mural nodules are rare in mucinous tumors. They are grossly and histologically distinct from the component of common mucinous tumors. From a practical point of view, mural nodules can be divided into two categories: benign and malignant. SLMN is the major form in the benign group whereas the most commonly reported nodules are composed of anaplastic carcinoma [3, 4, 13, 14]. Few cases have been reported as pure sarcomas yet [9, 15, 16]. The differential diagnosis between SLMN and malignancy (carcinoma or sarcoma) is critical because of their great prognostic difference [3]. Patients with mural nodules of pleomorphic carcinoma and/or high grade sarcoma are usually required for aggressive adjuvant treatment. Herein, we reported the morphology, immunophenotype, and molecular alterations in 3 patients with different types of mural nodules. This study adds some new knowledge on the pathogenesis of mucinous tumors with mural nodules.

Patient 1 was diagnosed as ovarian mucinous adenocarcinoma with mural nodules of anaplastic carcinoma according to the morphology and immunostaining profile. Mucinous tumors with carcinomatous mural nodules tend to occur in old patients, but are not exceptional in young girls before 20 years [14]. They were supposed to have a poor prognosis with a 50% mortality rate despite of the adjuvant chemotherapy and/or radiotherapy [3]. However, Provenza et al. [14] suggested that mural nodules of pleomorphic carcinomas were not essentially associated with poor clinical outcomes in patients with stage Ia tumors because all ten patients with unruptured tumors in their study were alive with no evidence of disease. Moreover, only 4 of 11 such patients in previous reports died of disease [14]. Our young patient died of disease at 44 months although she presented with a stage Ia tumor. The adverse clinical outcome may highlight the necessity of aggressive adjuvant therapy in patients with anaplastic mural nodules who were treated with fertility-sparing surgery.

Patient 1 demonstrated a histopathologic continuum that included benign, borderline, frankly invasive carcinoma and anaplastic carcinoma (mural nodules). Provenza et al. [14] showed that all 34 cases with pleomorphic carcinomas had at least mucinous borderline tumors and most carcinomas. The spindle cell morphology in the mural nodules may raise the concern about a sarcoma. However, a transition to carcinomas and the strong immunoreactivity for CK and CK7 were indicative of the carcinomatous nature. The morphological continuum and transition between mucinous tumors and pleomorphic carcinoma suggest that both components might be clonally related, and pleomorphic carcinoma may simply represent dedifferentiation or progression from mucinous tumors. Dedifferentiation is well-established in soft tissue and bone tumors, and more recently in epithelial tumors. Dedifferentiation is not uncommon in uterine or ovarian endometrioid carcinoma, and typically associated with an aggressive clinical course [17]. The dedifferentiation conception in anaplastic nodules is supported by our molecular and immunohistochemical analysis. The most frequent genetic alterations in ovarian mucinous tumors are somatic *KRAS* mutations with a prevalence of 50 to 68% in borderline and malignant tumors generally [6–8]. We found that patient 1 had an identical *KRAS* mutation (c.35G > A p.G12A) in both components in keeping with two previous studies [5, 10]. In addition, Mesbah Ardakani et al. [10] suggested that anaplastic mural carcinoma nodules might be more likely to arise in *KRAS* mutant tumors since 6 of 7 cases harbored somatic *KRAS* mutations. We observed aberrant p53 expression in the anaplastic carcinomatous nodules and wild-type expression in the mucinous tumors. Mutant-type p53 expression in anaplastic nodules indicates the potential role of further genomic alterations in an anaplastic morphologic phenotype in some cases. Similarly, Mesbah Ardakani et al. [10] showed that *p53* mutations were identified only in the anaplastic mural nodules in 2 of their cases. A recent study showed that aberrant p53 staining was exclusively in the undifferentiated component of the dedifferentiated endometrial carcinomas with neuroendocrine features; therefore, *p53* mutation was probably crucial for the maintenance of undifferentiated phenotype [18].

Patient 2 represented a rare true sarcomatous mural nodule in mucinous tumors. The distinct myxoid alterations raised the differential diagnosis among a variety of malignancies with similar changes such as myxoid endometrioid stromal sarcoma, myxoid leiomyosarcoma, myxoid liposarcoma, malignant nerve sheath tumor, inflammatory myofibroblastic tumor, and endometrioid stromal sarcoma [19]. The nodule might be best interpreted as endometrioid stromal sarcoma with myxoid changes because of strong CD10 staining, and lack of immunoreactivity for markers of epithelium, smooth muscles, skeletal muscles, nerves, endothelium, and melanoma. Nevertheless, CD10 is not a very specific marker for endometrial stromal tumors. No *PHF1* and *JAZF1* break-apart signals did not support the diagnosis of low grade endometrioid stromal sarcoma, in which, more than 50% cases harbor gene fusions [20]. *ZC3H7B-BCOR* high-grade endometrial stromal sarcoma is a recently characterized entity mimicking myxoid leiomyosarcoma morphologically [21]. Our case had similar pathological features, but the absence of *BCOR* break-apart signals and cyclin D1 staining opposed the diagnosis of *ZC3H7B-BCOR* sarcoma. A variety of sarcomatous nodules have been reported in 10 patients with ovarian mucinous tumors, such as osteosarcoma, fibrosarcoma, rhabodomyosarcoma, undifferentiated sarcoma, and high grade sarcoma (no other specified) [9, 15, 16]. By definition, the coexistence of mucinous adenocarcinoma and sarcoma in our patient could be interpreted as a carcinosarcoma. However, this designation is not preferred here since ovarian carcinosarcomas mostly harbor high grade serous carcinoma, frequently mixed with the sarcomatous component (usually high grade) that does not form a well-circumscribed nodule. The patient underwent left inferior lobectomy due to a subsequent pulmonary metastatic sarcoma, but she was alive with no evidence of disease for 40 months at present. Stage I low grade sarcomas, such as endometrial stromal sarcomas, have a relatively favorable prognosis despite a high frequency of tumor recurrence including lung metastasis whereas stage I primary ovarian well differentiated mucinous adenocarcinoma harbors an excellent prognosis with a high 10-year cause-specific survival (92.7%) [22–24]. We believe that the prognosis in our patient may be more closely associated with the sarcomatous components.

The molecular histogenesis of sarcomatous mural nodules in ovarian mucinous tumors has not well been investigated yet. Only one previous study reported that different *KRAS* mutations in a case of primary ovarian mucinous adenocarcinoma with mural nodule of high grade sarcoma [9]. In that study, the presence of *KRAS* mutations suggested that the sarcomatous component represented a dedifferentiated form of mucinous adenocarcinoma mostly likely. Contradictory to these findings, our molecular investigation on patient 2 observed no somatic mutations in the hotspots of *KRA S*, *AKT1*, *HRAS*, and *PI3KCA* in both carcinomatous and sarcomatous components. The morphological and immunohistochemical features indicated that the sarcomatous nodule in our case should be a true sarcoma rather than dedifferentiation from mucinous carcinoma. As a collar, the sarcomatous nodules and mucinous tumors most likely represent a tumor collision phenomenon. This hypothesis is clearly supported by LOH at different loci between both components.

SLMN in patient 3 should be discriminated from pleomorphic (spindle cell) carcinoma and true sarcoma. The well circumscription of the nodule, lack of necrosis and vascular invasion, absent or weak expression of multiple epithelial markers and lack of mutations in *KRAS*, *AKT1*, *HRAS*, and *PI3KCA* hotspots could aid in the diagnosis of SLMN. The patient received fertility-conserving surgery but no adjuvant therapy. She has been alive without evidence of disease at present. The unremarkable clinical course is also indicative of SLMN despite the relatively short follow up period. An earlier study found that 75% (6/8) patients were alive without evidence of disease at a mean follow-up interval of 12 years and the other two died of other causes [3]. SLMN may represent a florid reaction of submesothelial mesenchymal cells to intramural hemorrhage or to the mucinous material of the cyst, which eventually becomes a reactive and self-limited pseudotumor, such as the so-called inflammatory pseudotumor or post-operative spindle cell nodule described elsewhere. Nevertheless, our patient 3 should be long-term followed up closely because of the worrisome histology of SLMN and our limited knowledge to date.

## Conclusions

We describe 3 different histotypes of mural nodules in the ovarian mucinous tumors. The identical *KRAS* mutation in both components of mucinous carcinoma and the mural nodules of anaplastic carcinoma provides additional evidence to support that some anaplastic mural nodules represent the dedifferentiation form of mucinous tumors in which *p53* mutation may play crucial roles. In contrast, some mural nodules of true sarcomas most likely represent an independent origin from mucinous tumors. SLMN is believed to be a reactive and benign lesion, but should be treated with caution and merits long-term follow-up. More cases are critically required to consolidate these findings.

## Data Availability

Not applicable.

## Abbreviations

CDX2: Caudal type homeobox 2
CEA: Carcinomatous embryonic antigen
CK: Cytokeratin
EMA: Epithelial membrane antigen
ER: Estrogen receptor
FISH: Fluorescence in situ hybridization
MSI: Microsatellite instability
LOH: Loss of heterozygosity
PAX8: Paired box 8
PR: Progesterone receptor
SLMN: Sarcoma-like mural nodules
SMA: α-smooth muscle actin
STR: Short tandem repeats
